# Genetic Polymorphisms of Vitamin D Receptor Gene are Associated with Cervical Cancer Risk in Northeastern Thailand

**DOI:** 10.31557/APJCP.2020.21.10.2935

**Published:** 2020-10

**Authors:** Sophida Phuthong, Wannapa Settheetham-Ishida, Sitakan Natphopsuk, Takafumi Ishida

**Affiliations:** 1 *Department of Physiology, Faculty of Medicine, Khon Kaen University, Khon Kaen, Thailand. *; 2 *Chulabhorn International College of Medicine, Thammasat University, Pathum Thani, Thailand. *; 3 *Department of Biological Sciences, Graduate School of Science, The University of Tokyo, Tokyo, Japan. *

**Keywords:** Genetic polymorphism, Vitamin D receptor, cervical cancer

## Abstract

**Objective:**

This study aimed to explore whether *VDR* polymorphisms (*Fok1, Apa1 *and *Taq1*) are associated to the cervical cancer in Thai population.

**Materials and methods:**

Subjects of 204 cervical cancer patient and 204 age-matched healthy control were enrolled in the case-control study. *VDR* polymorphisms were detected by using real-time PCR. Haplotype analysis of three loci was applied to the obtained genotypes.

**Results:**

Significantly increased risk for cervical cancer was observed in carriers of TT genotype (p = 0.0388) and T allele (p = 0.0357) of *Fok1* and TC genotype (p = 0.0001), CC genotype (p = 0.0160) and the C allele of *Taq1* (p = 0.0001). Haplotype analyses revealed a significant correlation between C-T-C, T-G-C and T-T-C haplotypes and elevated risk for cervical cancer (OR = 2.06; 95%CI = 1.06-4.00; p = 0.0313, OR = 2.15; 95%CI = 1.22-3.80; p = 0.0078 and OR = 2.81; 95%CI = 1.53-5.16; p = 0.0006, respectively). Furthermore, haplotype carrying C allele of *Taq1* (C-G-C + C-T-C + T-G-C + T-T-C) significantly increased cervical cancer risk with OR of 1.92 (95%CI = 1.32-2.79, p = 0.0006).

**Conclusion:**

Our finding revealed an association between *VDR* polymorphisms and cervical cancer risk. *Taq1* C allele might be a molecular marker for cervical cancer development.

## Introduction

Cervical cancer is currently one of the most common cancers in women worldwide (Bray et al., 2018). The underlying causes for cervical cancer are human papillomavirus (HPV) infection, genetic variation and environmental factors (Natphopsuk et al., 2012; Wang et al., 2010). In addition, there is several lines of evidence suggesting the role of calcitriol (1,25-dihydroxyvitamin D3), active form of vitamin D, in female reproductive diseases including cervical cancer (Deuster et al., 2017; Reis et al., 2017; Vahedpoor et al., 2017). Vitamin D deficiency has been found to be associated with an increased risk of cancer (Tworoger et al., 2007).

Calcitriol, the biologically most potent form of vitamin D, exerts an antioncogenic effect, which mediates through various mechanisms; inhibition of proliferation, promotion of cell differentiation and apoptosis (Dou et al., 2016; Samuel and Sitrin, 2008). Calcitriol activity is controlled by binding of calcitriol to vitamin D receptor (VDR) which is then translocated into the nucleus, where it forms a calcitriol-VDR/retinoid X receptor (RXR) complex. Consequently, this complex binds to vitamin D response elements (VDREs) on the target genes which then regulates the transcription (Pike and Meyer, 2012). In view of this control mechanism, calcitriol action thus depends on VDR activity (Uitterlinden et al., 2004).

The VDR protein is encoded by the *VDR* gene which is found in chromosome 12q13.11. This gene is expressed in normal and cancerous cervix, indicating the anti-tumor effects of calcitriol on the cervix (Deuster et al., 2017; Kaabachi et al., 2014; Reichrath et al., 1998). However, VDR activity may be influenced by *VDR* genetic polymorphisms (Köstner et al., 2009), several of which have been identified. The frequently studied *VDR* polymorphisms such as *Fok1, Apa1* and *Taq1*, all involving transitions (C>T in exon 2), (G>T in intron 8) and (T>C in exon 9), respectively (Köstner et al. 2009; McCullough et al. 2007; Wang et al. 2013). *Fok1* produces an alternative transcription initiation site in which the T allele generates the VDR protein with three amino acid longer (427 amino acids) than the protein produced by C allele (424 amino acids). The longer protein exerts less transactivation capacity (Amadori et al., 2017). The *Apa1* and *Taq1* polymorphic sites are close to the 3’ untranslated region (3’-UTR) of *VDR* mRNA which affects mRNA stability and the post-transcriptional process (Guo et al., 2018; Whitfield et al., 2001). Interestingly, *Fok1* have been shown to be linked with each other of 3’UTR indicating their interaction effects in modulating VDR activity (Israni et al., 2009; Kaabachi et al., 2014). As a result, most of studies have performed not only *VDR* genotype but also haplotype analysis in relation to cancer susceptibility but the results were inconsistent (Flügge et al., 2007; Kaabachi et al., 2014; Onen et al., 2008).

The study of *VDR* genetic polymorphisms is one approach to understand interindividual susceptibility to cervical cancer which have not been evaluated previously. Hence, this study provides the first report regarding the analysis of *VDR* polymorphisms (*Fok1, Apa1* and *Taq1*) individually and *VDR* haplotype on cervical cancer risk among Thai population.

## Materials and Methods

*Study subjects*

The female volunteers were recruited from Srinagarind hospital, Khon Kaen University and Khon Kaen hospital, Khon Kaen, Thailand. Participants were divided into two groups that included 204 patients with pathologically diagnosed squamous cell carcinoma of the cervix (SCCA) and 204 age-matched healthy controls (5-years interval). Informed consent was obtained from all participants after which peripheral blood leukocytes were collected for *VDR* polymorphism analysis. The study was approved by the Ethic Committee of Khon Kaen university (Reference No. HE621103).

*Detection of VDR polymorphisms*

Genomic DNA (gDNA) was extracted from buffy coat using GF-1 Blood DNA Extraction Kit (Vivantis, USA). Real-time PCR with Taqman^®^ probe (Applied Biosystems, USA) was used for *Fok1* (*rs2228570*), *Apa1 *(*rs7975232*) and *Taq1* (*rs731236*) detection. 

The real-time PCR conditions involved: a holding stage at 95^o^C for 10 minutes, followed by 40 cycles each of denaturation at 95^o^C for 15 seconds, annealing and extension at 60^°^C for 60 seconds. 

*Statistical analyses*

Statistical analyses were performed using the STATA software. Deviation from Hardy-Weinberg equilibrium (HWE) for the genotype of each loci was tested with the Pearson’s chi-square (*χ2* test). The haplotypes were inferred using PHASE algorithm version 2.1.1. Association between genotypes or haplotypes of *VDR* and cervical cancer risk was tested by calculating the odd ratios with (OR) 95% confidence intervals (95%CI) using logistic regression analyses. A two-tailed p-value of less than 0.05 was considered to be statistically significant. 

## Results

Table 1 presents association between *VDR* gene polymorphisms and cervical cancer risk. Genotype distribution of *VDR (Fok1*, *Apa1* and *Taq1)* polymorphisms among controls were consistent with HWE (p > 0.05). For the *Fok1* and *Taq1*, allele frequencies were found to differ significantly between the patients and the controls (p < 0.05).

Interestingly, the TT genotype and the T allele of *Fok1* was significantly associated with increased risk for cervical cancer (crude OR = 1.79; 95%CI = 1.03-3.11; p = 0.0388 and adjusted OR = 2.66; 95%CI = 1.17-6.04; p = 0.020 for TT vs. CC and OR = 1.34; 95%CI = 1.02-1.77; p = 0.0357 for T vs. C). For *Taq1* locus, TC and CC genotypes and the C allele significantly increased cervical cancer risk (crude OR = 2.48; 95%CI = 1.59-3.87; p < 0.0001 and adjusted OR = 2.36; 95%CI = 1.28-4.35; p = 0.006 for TC vs. TT and crude OR = 2.44; 95%CI = 1.10-5.58; p = 0.0160 and adjusted OR = 2.11; 95%CI = 0.73-6.10; p = 0.167 for CC vs. TT and OR = 1.95; 95%CI = 1.42-2.67; p < 0.0001 for C vs. T), whereas *Apa1 *was not associated with cervical cancer risk (p > 0.05).

Haplotype (*Fok1-Apa1-Taq1*) analysis regarding cervical cancer risk is presented in Table 2 where C-G-T was found to be the most common in both patients and controls. C-T-C, T-G-C and T-T-C haplotypes significantly increased the risk for cervical cancer compared to C-G-T haplotype with OR = 2.06; 95%CI = 1.06-4.00; p = 0.0313, OR = 2.15; 95%CI = 1.22-3.80; p = 0.0078 and OR = 2.81; 95%CI = 1.53-5.16; p = 0.0006, respectively. Interestingly, an elevated risk for cervical cancer was observed among haplotypes carrying C allele compared to T allele of *Taq1*; C-G-C vs. C-G-T (OR = 1.05; 95%CI = 0.57-1.92; p = 0.9334), C-T-C vs. C-T-T (OR = 3.22; 95%CI = 1.47-7.05; p = 0.0030), T-G-C vs. T-G-T (OR = 2.00; 95%CI = 1.12-3.56; p = 0.0181 and T-T-C vs. T-T-T (OR = 2.62; 95%CI = 1.31-5.25; p = 0.0059). Furthermore, carriers of *Taq1* C haplotype (C-G-C + C-T-C + T-G-C + T-T-C) had 1.92 times higher risk for cervical cancer compared to C-G-T haplotype (95%CI = 1.32-2.79, p = 0.0006).

**Table 1:** Association between *VDR *Polymorphisms and Risk for Cervical Cancer

Genotypes/Alleles	Control n (%)	Case n (%)	Crude OR [95%CI, *p*]	Adjusted OR^b^ [95%CI, *p*]
*Fok1* ^a^				
CC	55 (29.6)	45 (22.06)	1	1
CT	106 (51.96)	96 (47.06)	1.11 [0.67-1.84, 0.6790]	1.44 [0.71-2.94, 0.313]
TT	43 (21.08)	63 (30.88)	1.79 [1.03-3.11, 0.0388*]	2.66 [1.17-6.04, 0.020*]
CT+TT	149 (73.04)	159 (77.94)	1.3 [0.81-2.11, 0.2498]	1.77 [0.90-3.46, 0.096]
C	0.53	0.46	1	1
T	0.47	0.54	1.34 [1.02-1.77, 0.0357*]	NA
*Apa1* ^a^				
GG	100 (49.02)	94 (46.08)	1	1
GT	83 (40.69)	82 (40.2)	1.05 [0.68-1.63, 0.8143]	1.11 [0.61-2.04, 0.728]
TT	21 (10.29)	28 (13.73)	1.42 [0.72-2.82, 0.2771]	1.96 [0.79-4.84, 0.144]
GT+TT	104 (50.98)	110 (53.92)	1.13 [0.75-1.69, 0.5520]	1.27 [0.72-2.24, 0.410]
G	0.69	0.66	1	1
T	0.31	0.34	1.16 [0.86-1.55, 0.3302]	NA
*Taq1* ^a^				
TT	133 (65.2)	88 (43.14)	1	1
TC	58 (28.43)	95 (46.57)	2.48 [1.59-3.87, 0.0001*]	2.36 [1.28-4.35, 0.006*]
CC	13 (6.37)	21 (10.29)	2.44 [1.10-5.58, 0.0160*]	2.11 [0.73-6.10, 0.167]
TC+CC	71 (34.8)	116 (56.86)	2.47 [1.62-3.76, 0.0001*]	2.31 [1.30-4.11, 0.004*]
T	0.79	0.66	1	1
C	0.21	0.34	1.95 [1.42-2.67, 0.0001*]	NA

OR, odds ratio, CI: confidence interval, * *p* < 0.05, NA: not applicable; ^a^ no deviation from Hardy–Weinberg equilibrium (*p* > 0.05 by χ2 test); ^b^ adjusted multiple logistic regression for partners’ smoking, contraceptive use and HPV infection

**Table 2:** Association between *VDR* Haplotypes (*Fok1-Apa1-Taq1*) and Risk for Cervical Cancer

*Fok1-Apa1-Taq1*	Control n (%)	Case n (%)	OR [95%CI, p]	OR [95%CI, p]
C-G-T	127 (31.13)	108 (26.47)	1	1
C-G-C	27 (6.62)	24 (5.88)	1.05 [0.57-1.92, 0.9334]	1.05 [0.57-1.92, 0.9334]
C-T-T	46 (11.27)	25 (6.13)	0.64 [0.37-1.11, 0.1094]	1
C-T-C	16 (3.92)	28 (6.86)	2.06 [1.06-4.00, 0.0313*]	3.22 [1.47-7.05, 0.0030*]
T-G-T	106 (25.98)	97 (23.77)	1.08 [0.74-1.57, 0.7026]	1
T-G-C	23 (5.64)	42 (10.29)	2.15 [1.22-3.80, 0.0078*]	2 [1.12-3.56, 0.0181*]
T-T-T	45 (11.03)	41 (10.05)	1.07 [0.65-1.76, 0.7847]	1
T-T-C	18 (4.41)	43 (10.54)	2.81 [1.53-5.16, 0.0006*]	2.62 [1.31-5.25, 0.0059*]
C-G-T	127 (31.13)	108 (26.47)	1	1
C-G-C + C-T-C + T-G-C + T-T-C	84 (20.59)	137 (33.57)	1.92 [1.32-2.79, 0.0006*]	1.92 [1.32-2.79, 0.0006*]

OR, odds ratio; CI, confidence interval; * *p* < 0.05

## Discussion

To our knowledge, this study is the first to report an association between *VDR* polymorphisms and cervical cancer risk in a Thai population. Increased risks for cervical cancer observed among carriers of the TT genotype and the T allele of *Fok1* polymorphism indicated this allele to be a susceptible risk allele. In agreement with these findings, the presence of the *Fok1* T allele was a risk allele for ovarian, breast and renal cancers (Arjumand et al., 2012; Liu et al., 2013; Wang et al., 2013). The *Fok1* T allele could decrease *VDR* transcription and translation efficacy which was reported to reduce anti-carcinogenic properties of calcitriol and contributes to an increased cancer susceptibility (Amadori et al., 2017; Chen et al., 2018; Feldman et al., 2014). Hence, this report confirms the role of *Fok1* T allele in raising cancer susceptibility.

Our finding of an absence of correlation between *Apa1 *polymorphism and cervical cancer risk agrees with reports of other studies in prostate, breast and ovarian cancers (Clendenen et al., 2008; Wang et al., 2016). The *Apa1 *polymorphism found in the intron 8 does not change the VDR amino acid sequence (Köstner et al. 2009). Therefore, Apa1 would be less expected to mimic VDR expression and function as seen in earlier study which provided evidence that this polymorphism does not affect VDR expression level (Selvaraj et al., 2009). 

Regarding *Taq1* polymorphism, our results indicate a signiﬁcant role of the C allele in the cervical cancer risk which was not previously observed in other cancers such as breast, prostate, and colorectal cancer (Bodiwala et al., 2004; Flügge et al., 2007; Gapska et al., 2009). A variant allele of *Taq1* locus has been shown to regulate VDR mRNA stability which alters expression and transactivation capacity (McCullough et al., 2009; Whitfield et al., 2001). This mechanism might alter the state of VDR in the cancer susceptibility (Chen et al., 2018; Serrano et al., 2016). To our knowledge, the C allele of *Taq1* might be a potential diagnostic biomarker for cervical cancer susceptibility. Nevertheless, these conflicting results provide a hypothesis that the influence of *VDR* on cancer susceptibility might depend on dietary and environmental factors that potentially influence calcitriol levels and VDR activity (Bodiwala et al., 2004; Köstner et al., 2009; McCullough et al., 2007). Hence, further studies should consider gene-environment interaction to clarify the role of calcitriol and *VDR* on cervical cancer susceptibility.

*VDR* haplotype (*Fok1-Apa1-Taq1*) analysis was performed to provide more conclusive information regarding genetic variation. In accordance with allele frequencies, C-G-T haplotype was predominant in the healthy Thai population as previously reported for another Asian population (Li et al., 2018).

Haplotypes containing the T allele of *Fok1* or C allele of *Taq1* (C-T-C, T-G-C or T-T-C) were associated with statistically increased cervical cancer risks compared to the most common C-G-T haplotype. Nevertheless, haplotypes carrying only *Fok1* T allele (T-T-C and T-T-T) were not correlated with increased cervical cancer risk, suggesting that the effect of *Fok1* polymorphism might be depending on the interaction with *Taq1* loci. To clarify the possible significance of *Taq1* in cervical cancer risk, *Taq1* pair haplotypes analysis was performed. The presence of *Taq1* C allele was found to be strongly associated with an increased risk for cervical cancer, which was in line with the result of *Taq1* allelic analysis. Moreover, an increased cervical cancer risk observed in the haplotype analysis was higher than the risk observed in each allele. Thus, the role of *VDR* genetic variation on cervical cancer susceptibility is most likely influenced by the presence of the C allele of *Taq1* which produced less VDR activity and less responsive to calcitriol.

In conclusion, this study is the first to demonstrate the role of *VDR* polymorphisms, especially *Taq1 *polymorphism in cervical cancer risk among Northeastern Thai women. Thus, the *Taq1* C allele might be considered an important molecular marker for cervical cancer risk that may predict possible cervical cancer development. 
